# Breaking Through Bottlenecks for Thermally Conductive Polymer Composites: A Perspective for Intrinsic Thermal Conductivity, Interfacial Thermal Resistance and Theoretics

**DOI:** 10.1007/s40820-021-00640-4

**Published:** 2021-04-13

**Authors:** Junwei Gu, Kunpeng Ruan

**Affiliations:** MOE Key Laboratory of Material Physics and Chemistry Under Extraordinary Conditions, Shaanxi Key Laboratory of Macromolecular Science and Technology, School of Chemistry and Chemical Engineering, Northwestern Polytechnical University, Xi’an, Shaanxi, 710072 P. R. China

**Keywords:** Thermally conductive polymer composites, Intrinsic thermal conductivity, Interfacial thermal resistance, Thermal conduction models, Thermal conduction mechanisms

## Abstract

Rapid development of energy, electrical and electronic technologies has put forward higher requirements for the thermal conductivities of polymers and their composites. However, the thermal conductivity coefficient (*λ*) values of prepared thermally conductive polymer composites are still difficult to achieve expectations, which has become the bottleneck in the fields of thermally conductive polymer composites. Aimed at that, based on the accumulation of the previous research works by related researchers and our research group, this paper proposes three possible directions for breaking through the bottlenecks: (1) preparing and synthesizing intrinsically thermally conductive polymers, (2) reducing the interfacial thermal resistance in thermally conductive polymer composites, and (3) establishing suitable thermal conduction models and studying inner thermal conduction mechanism to guide experimental optimization. Also, the future development trends of the three above-mentioned directions are foreseen, hoping to provide certain basis and guidance for the preparation, researches and development of thermally conductive polymers and their composites.

## Highlights

- Bottlenecks in the field of thermally conductive polymer composites are raised, and corresponding reasons are analysed.
- Three possible directions for breaking through such bottlenecks are put forward, and current advances in these three directions are illustrated.
- Future development trends and demands are foreseen to help the development of thermally conductive polymers and their composites.

## Graphical abstract

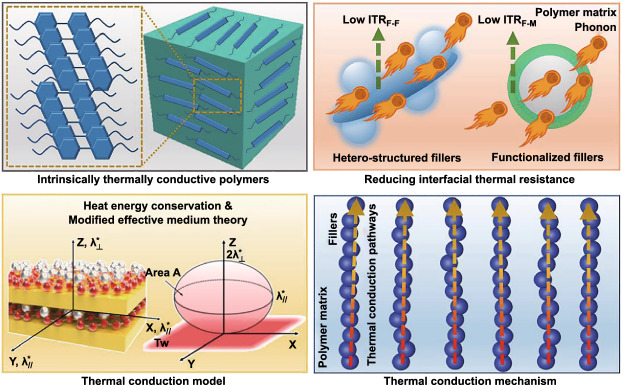

## Introduction

With the rapid development of energy, electrical and electronic technologies, the rapid accumulation of heat in related equipment and components will inevitably result in the serious threat to their stabilities and reliabilities [1]. Polymers are frequently used in energy, electrical and electronic fields due to their light weight, high specific strength/modulus, easy processing, excellent chemical stability and low cost [2]. However, the thermal conductivity coefficient (*λ*, 0.18 ~ 0.44 W m^−1^ K^−1^) values of polymers are often low, which cannot meet the requirements of highly efficient and fast thermal conduction/dissipation for organic solar cells, energy storage materials, UHV power transmission equipment and high-power LEDs [3]. Therefore, the researches and development of polymers and their composites with high thermal conduction/dissipation capabilities and excellent mechanical properties have urgent theoretical significance and practical application values for the design and expansion of materials in the fields of energy, electrical and electronic technologies.

Thermally conductive polymers can be divided into two types according to the preparation process: intrinsic type and filled type [4]. Intrinsically thermally conductive polymers are obtained via special physical structures (such as orientation, liquid crystalline and crystalline structure) by changing the structures of polymer chain units in the process of polymer synthesis and processing, in order to improve the intrinsic thermal conductivities of the polymers. Filled-type thermally conductive polymer composites are fabricated by adding highly thermally conductive fillers into the polymer matrix, and thereafter giving excellent thermal conductivities to the polymers by directly physical blending.

Till present, many researchers have prepared a variety of thermally conductive polymers and their composites through the two above-mentioned methods, but in the end, most of *λ* values are still difficult to meet expectation, which has become the major bottleneck in this field [5]. Our research group has long focused on the controllable fabrication and inner mechanisms of thermally conductive polymers and their composites. Based on the intrinsic high thermal conductivities, blending, and compounding and external field-induced processing, the thermal conduction properties of “polymers-interfaces-fillers” and constitutive relationships between “molecular chains-thermal conduction pathways-thermal conductivities” have been investigated, a series of thermally conductive polymer composites and products have been prepared, and the thermal conduction mechanisms have also been improved. Based on the previous research experiences, this paper proposes the research ideas and directions that can be taken in the future for breaking through the bottlenecks in the field of thermally conductive polymer composites, so as to provide a certain basis and guidance for the preparation, research, and development of thermally conductive polymers and their composites.

## Possible Directions for Breaking through Bottlenecks for Thermally Conductive Polymer Composites

### Intrinsically Thermally Conductive Polymers

One of the most important reasons why the *λ* values of thermally conductive polymer composites are difficult to achieve the expectation is that the intrinsic *λ* values of polymers are low. Therefore, even if polymers are filled with thermally conductive fillers with very high *λ*, the improvements of *λ* are still limited. Studies have shown that when the ratio of the *λ* values of the polymer matrix to the thermally conductive fillers is less than 1:100, it is difficult to efficiently improve the thermal conductivities of polymer composites by only filling thermally conductive fillers with high *λ* [6]. As a consequence, it is very critical to improve the intrinsic thermal conductivity of the polymer matrix, and the preparation of synthetic intrinsically thermally conductive polymers through molecular design is a novel idea and direction to improve the thermal conductivities of polymers and their composites.

Researches on intrinsically thermally conductive polymers firstly began in Takezawa’s research group in Japan. Based on the orderly structure of the molecules, the liquid crystalline epoxy monomers with biphenyl groups were synthesized, and the *λ* of the cured epoxy exceeded 0.90 W m^−1^ K^−1^, 5 times that of conventional epoxy resin (0.18 W m^−1^ K^−1^) [7]. However, it should be noted that the *λ* value was measured and calculated by the AC calorimetry method, not comparable with the existing *λ* test equipment, such as the Linseis THB (Germany), AB Hot Disk (Sweden) and Netzsch LFA467 (Germany) by heat flow method, plane transient method and laser flash method. Jeong et al*.* [8] prepared a kind of side-chain epoxy resin containing cyanobiphenyl mesogenic end groups. After curing and crosslinking by diamine, the liquid crystalline epoxy resin still retained the oriented liquid crystalline region, whose *λ* could reach 0.46 W m^−1^ K^−1^, because the microstructure contained anisotropic molecular orientation. In the previous work of our research group [9], we designed and synthesized side-chain liquid crystalline epoxy (S-LCE), which was prepared by thiol-epoxide nucleophilic ring-opening reaction and coating method. Intrinsically highly thermally conductive and self-healing liquid crystalline epoxy films (LCEF) exhibited excellent intrinsic thermal conductivities and self-healing capabilities, whose through-plane *λ* (*λ*_*⊥*_) and in-plane *λ* (*λ*_*∥*_) were 0.33 and 1.25 W m^−1^ K^−1^, respectively, much higher than *λ*_*⊥*_ (0.19 W m^−1^ K^−1^) and *λ*_*∥*_ (0.65 W m^−1^ K^−1^) of general bisphenol A epoxy resin (E-51). Furthermore, Gu et al*.* [10] designed and synthesized a kind of liquid crystalline epoxy based on biphenyl mesogens, using 4, 4′-diaminodiphenylmethane (DDM) as curing agent, to fabricate cured epoxy resin (LCER) with *λ* of 0.51 W m^−1^ K^−1^, about 2.7 times that of conventional E-51 epoxy resin (0.19 W m^−1^ K^−1^). When the amount of BN was 30 wt%, the corresponding *λ* of thermally conductive BN/LCER composites was 1.02 W m^−1^ K^−1^, which was much higher than that of thermally conductive BN/E-51 composites (0.52 W m^−1^ K^−1^) with the same fillers amount, proving that preparing intrinsically thermally conductive polymers is a practical and feasible strategy to effectively improve the *λ* of thermally conductive polymer composites.

**Fig. 1:**
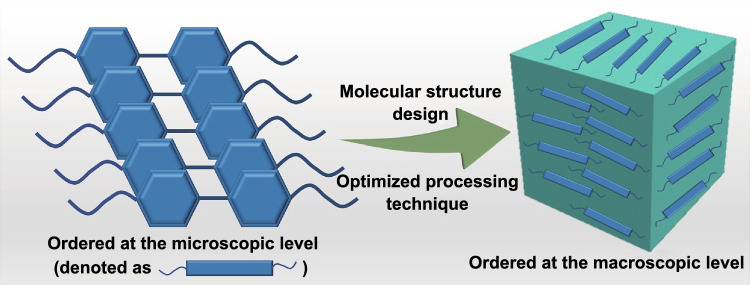
Schematic diagram of perspective intrinsically thermally conductive polymers with ordered structures at both microscopic and macroscopic levels

However, the intrinsically thermally conductive polymers prepared at present are mainly concentrated in epoxy resins, which are relatively single in types. There are few reports on intrinsically thermally conductive polyimide (PI), polyoxymethylene (POM), polycarbonate (PC) or other high-performance engineering polymers. In addition, the chains of liquid crystalline polymers are usually only partially ordered at the microscopic level, and still present the isotropically disordered state at the macroscopic level, which affects the efficient improvement of intrinsic thermal conductivities. In the future research works on intrinsically thermally conductive polymers, researchers can prepare and synthesize intrinsically thermally conductive high-performance engineering polymers to broaden the application ranges of intrinsically thermally conductive polymers. It is also an excellent direction to improve the macroscopy order of the polymer chains by reasonable molecular structure design and optimized processing technique (Fig. 1), in order to further greatly enhance the intrinsic thermal conductivities of polymers.

### Interfacial Thermal Resistance in Thermally Conductive Polymer Composites

Interfaces play an important role and have great influences on *λ* in thermally conductive polymer composites [11]. During the thermal conduction process, vibration harmonic, acoustic & modulus mismatch occur for phonon at the interfaces, thus resulting in severe scattering and causing the mean free path of phonon to drop dramatically [12]. Corresponding macroscopic evidence is that when the heat flow passes through the interface, it is often obstructed to a certain extent, causing serious heat loss, and then reducing the *λ* of polymer composites [13]. Therefore, it will be the key idea to improve the interfaces in thermally conductive polymer composites and to reduce interfacial thermal resistance (ITR), so as to further improve the *λ* of polymer composites. The interfaces include those between thermally conductive fillers and polymer matrix and those between different types of thermally conductive fillers. The corresponding ITR are denoted as ITR_F-M_ and ITR_F-F_ in this paper.

**Fig. 2:**
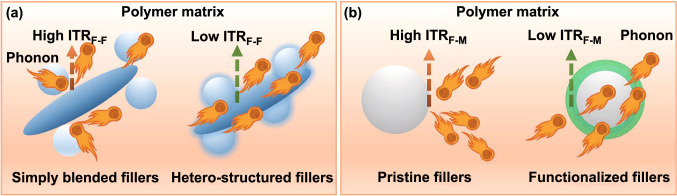
Schematic diagram of ITR_F-F_ (**a**) and ITR_F-M_ (**b**)

Researchers reported that the fabrication of thermally conductive fillers with hetero-structures can effectively decrease the ITR_F-F_ (Fig. 2a). Zou et al*.* [14] coated the surface of alumina (Al_2_O_3_) with boron nitride nanosheets (BNNS) to fabricate hetero-structured Al_2_O_3_@BNNS thermally conductive fillers, and then prepared thermally conductive Al_2_O_3_@BNNS/epoxy composites. When the volume ratio of BNNS to Al_2_O_3_ was 1:7 and the amount of Al_2_O_3_@BNNS was 65 vol%, the *λ* of thermally conductive Al_2_O_3_@BNNS/epoxy composites reached 2.43 W m^−1^ K^−1^, higher than pure epoxy resin (0.21 W m^−1^ K^−1^), single Al_2_O_3_/epoxy (1.39 W m^−1^ K^−1^) and simply blended (Al_2_O_3_/BNNS)/epoxy (1.94 W m^−1^ K^−1^) composites under the same fillers amount. In the previous work of our research group, Gu et al*.* [15] fabricated hetero-structured silicon carbide-BNNS (SiC-BNNS) thermally conductive fillers by sol–gel & *in-situ* growth method. When the mass ratio of SiC to BNNS was 1:1 and the total amount was 20 wt%, the corresponding *λ* of thermally conductive SiC-BNNS/epoxy composites was as high as 0.89 W m^−1^ K^−1^, higher than single SiC/epoxy (0.43 W m^−1^ K^−1^), single BNNS/epoxy (0.61 W m^−1^ K^−1^) and simply blended (SiC/BNNS)/epoxy (0.52 W m^−1^ K^−1^) composites, which proved that fabrication of thermally conductive fillers with hetero-structures can improve the interfaces between different types of fillers, reduce the phonon scattering at the interfaces and decrease ITR_F-F_.

In order to reduce the ITR_F-M_, researchers usually functionalize the surfaces of thermally conductive fillers (Fig. 2b). Guo et al*.* [16] functionalized the surfaces of multi-wall carbon nanotubes by triethoxyvinylsilane (s-MWCNTs). When the amount of s-MWCNTs was 10 wt%, the *λ* of thermally conductive s-MWCNTs/poly(vinylidene fluoride) (PVDF) composites was 1.55 W m^−1^ K^−1^, which was about 9 times that of pure PVDF, and also higher than that of thermally conductive pristine MWCNTs/PVDF composites (0.48 W m^−1^ K^−1^). In the previous work of our research group, Gu et al*.* [17] used polydopamine (PDA) to functionalize the surfaces of BNNS to prepare BNNS@PDA thermally conductive fillers. When the amount of BNNS@PDA was 50 wt%, the corresponding *λ*_*⊥*_ and *λ*_*∥*_ of thermally conductive BNNS@PDA/aramid nanofiber (ANF) composite papers reached 0.62 and 3.94 W m^−1^ K^−1^, respectively, which were higher than pure ANF paper (*λ*_*⊥*_ = 0.22 W m^−1^ K^−1^, *λ*_*∥*_ = 1.33 W m^−1^ K^−1^) and thermally conductive pristine BNNS/ANF composite papers (*λ*_*⊥*_ = 0.52 W m^−1^ K^−1^, *λ*_*∥*_ = 3.33 W m^−1^ K^−1^). Calculation based on the improved Hashin–Shtrikman model [18] showed that the surface functionalization of BNNS could effectively reduce the in-plane and through-plane ITR_F-M_ values from 0.1644 and 0.1696 to 0.1590 and 0.1587, respectively. In addition, Gu et al*.* [19] prepared thermally conductive aminated reduced graphene oxide (NH_2_-rGO)/PI composite films. When the amount of NH_2_-rGO was 15 wt%, the *λ*_*⊥*_ and *λ*_*∥*_ of thermally conductive NH_2_-rGO/PI composite films reached 0.74 and 7.13 W m^−1^ K^−1^, respectively, higher than pure PI film (*λ*_*⊥*_ = 0.21 W m^−1^ K^−1^, *λ*_*∥*_ = 0.87 W m^−1^ K^−1^) and thermally conductive pristine rGO/PI composite films (*λ*_*⊥*_ = 0.62 W m^−1^ K^−1^, *λ*_*∥*_ = 5.50 W m^−1^ K^−1^). Using Raman spectroscopy, the internal ITR_F-M_ and phonon scattering at the interfaces were successfully characterized, revealing the interfacial thermal conduction mechanism, showing the inner reason for effectively reducing the ITR_F-M_ and improving the *λ* of thermally conductive polymer composites from the microscopic perspective.

However, most of the domestic and foreign researches on ITR are not in-depth enough. The decreases in ITR are indirectly reflected by the increases in *λ*, and there is a lack of universal mathematical models, test methods and related measurement equipment for ITR. In the future research works on ITR, researchers would try to establish more universal mathematical models on ITR for the new forms and characteristics of thermally conductive polymer composites, and speed up the in-depth cooperation with thermal properties measurement companies, quickly develop the multi-system applicable and highly universal ITR test methods and measurement equipment.

### Thermal Conduction Models and Inner Mechanisms of Thermally Conductive Polymer Composites

**Fig. 3:**
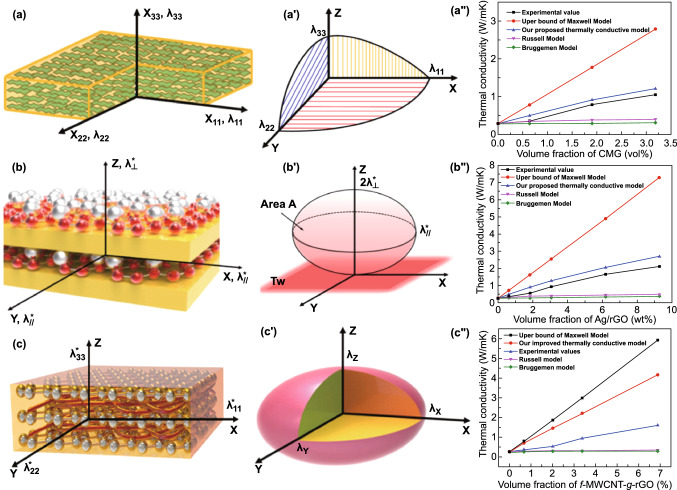
Schematic diagrams of proposed thermal conduction models and comparison with traditional models for thermally conductive CMG/PI (**a–a′′**) [24], Ag/rGO/PI (**b–b′′**) [25] and f-MWCNTg-rGO/PI (**c–c′′**) [26] composites

The *λ* values of the thermally conductive polymer composites are closely related to the intrinsic *λ* of polymers, the type and amount of thermally conductive fillers, as well as ITR. Studying the thermal conduction models will help to clarify the influencing factors theoretically, and calculate and predict the *λ* of composites in specific system. Researchers have proposed a variety of thermal conduction models, among which Y. Agari’s [20], Maxwell-Eucken’s [21] and Nielsen-Lewis’ models [22] are more successful. However, the existing thermal conduction models have narrow application range, not taking into account the shape, amount and surface properties of thermally conductive fillers, as well as ITR, etc., so that there are always certain errors between the predicted *λ* values by models and the experimental values. In our previous works, we optimized the classic series & parallel thermal conduction models for thermally conductive BN/cyanate ester composites, which had better *λ* fitting degree than other classic thermal conduction models [23]. Also, for thermally conductive carbon-based fillers/PI composites, based on the modified effective medium theory and the principle of heat energy conservation, thermal conduction models suitable for anisotropic composites were proposed, showing better *λ* fitting degree than other classical thermal conduction models (Fig. 3) [24–26]. Moreover, COMSOL Multiphysics software was used to establish models to simulate the thermal conduction process of thermally conductive epoxy laminated composites, and the simulation results had a high degree of matching with the experimental results [27]. In the future research works on the thermal conduction models, researchers need to fully consider more practical influencing factors, quantify and introduce these factors into the thermal conduction models, and improve the degree of matching between the thermal conduction models and the experimental results.

**Fig. 4:**
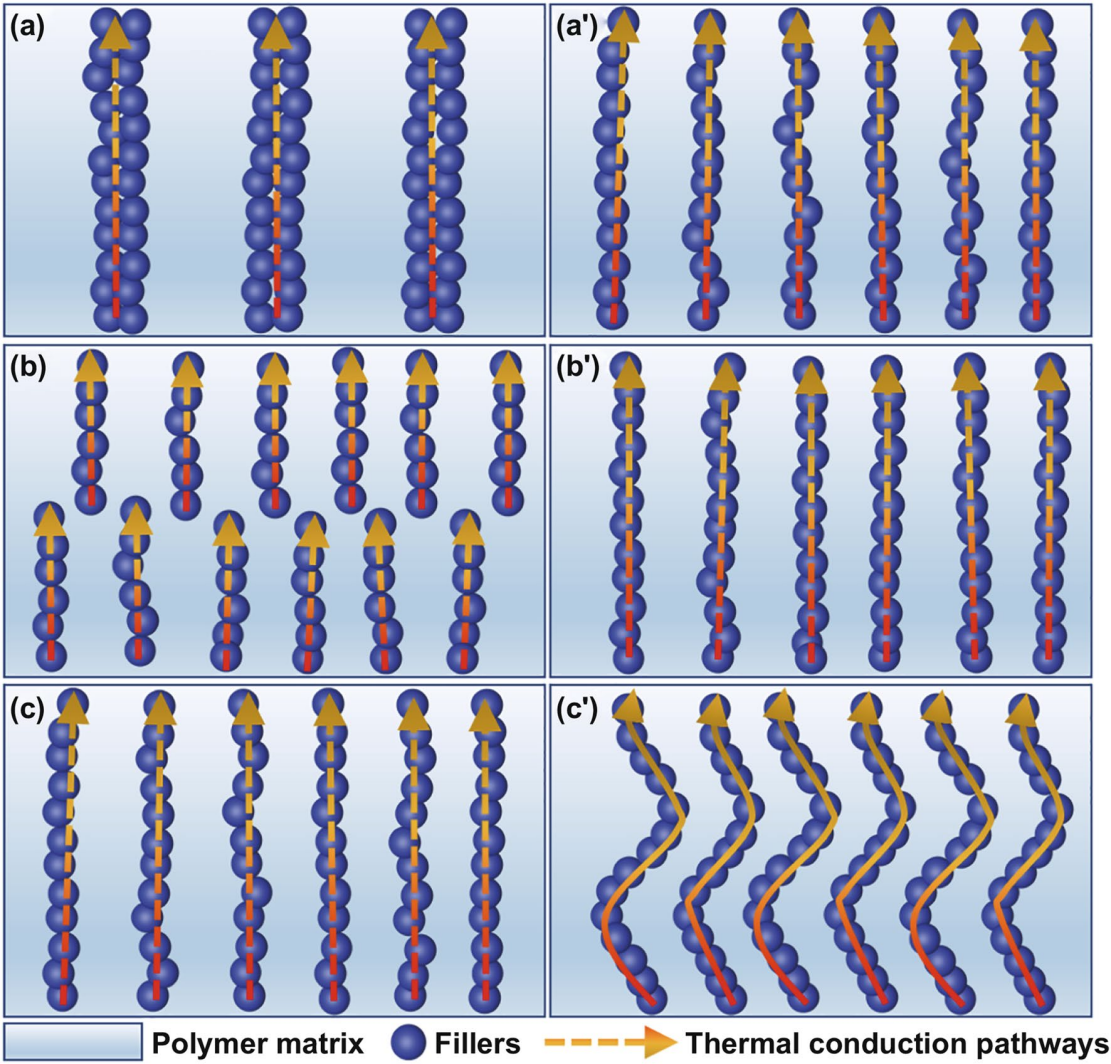
Thermal conduction pathways in different numbers (fewer-**a** & more-**a′**), different continuity (discontinuous-**b** & continuous-**b′**) and different length and shapes (short and straight-**c** & long and curved-**c′**)

Regarding the inner thermal conduction mechanisms of thermally conductive polymer composites, it is generally recognized internationally that it is based on the thermal conduction pathways formed by thermally conductive fillers [28]. However, whether the final *λ* values of the thermally conductive polymer composites are proportional to the number of thermal conduction pathways in the composites needs to be further verified (Fig. 4a–a′), and what form and length of thermal conduction pathways (continuous or discontinuous pathways, long or short pathways, straight or curved pathways, etc*.*) will determine *λ* values also needs further consideration (Fig. 4b–c′). Also, the percolation behaviour of thermal conduction has an extremely important impact on the rapid *λ* improvement of polymer composites. Based on the researches on the constitutive relationship of “thermal conduction pathways-thermal conductivities” for thermally conductive graphite nanoplatelets/polyphenylene sulphide (GNPs/PPS) composites, our research group proposed that the GNPs/PPS composites system showed thermal conduction percolation behaviour, but the behaviour mostly existed in high-*λ* carbon-based fillers (such as GNPs, carbon nanotubes (CNT) and graphene) [29]. However, the physical properties of the thermal conduction percolation behaviour are still the question worth discussing, due to that, with the same fillers, the increase in thermal conductivity is much lower than that in electrical conductivity. Therefore, in the future research works on the thermal conduction mechanisms of thermally conductive polymer composites, it is necessary to conduct in-depth analysis and exploration on the formation approaches, methods and degrees of thermal conduction pathways in the thermally conductive polymer composites, as well as the thermal conduction percolation behaviour, in order to develop the thermal conduction mechanisms of thermally conductive polymer composites, and ultimately guide the optimization of experiments and production.

## Summary and Perspectives

In summary, although progresses have been made in thermally conductive polymer composites, their *λ* values are mostly still lower than expected. Aimed at that, based on the accumulation of the previous research works by related researchers and our research group, this paper proposes three possible directions to break through the bottlenecks: (1) preparing and synthesizing intrinsically thermally conductive polymers, (2) reducing the ITR in the thermally conductive polymer composites and (3) establishing suitable thermal conduction models and studying inner thermal conduction mechanisms to guide experimental optimization. Also, the future development trends of the three above-mentioned directions are foreseen, hoping to provide certain basis and guidance for the preparation, researches and development of thermally conductive polymers and their composites. It is believed that after breaking through the current bottlenecks, thermally conductive polymer composites, as the basic support for the development of human society, with further assistance by intellectualization of the materials through active heat dissipation, will show irreplaceable roles in various aspects such as aerospace, energy management, artificial intelligence, new energy, high-end equipment manufacturing and energy-efficient electronic devices.

## References

[1] Zhang F, Feng Y, Feng W (2020). Three-dimensional interconnected networks for thermally conductive polymer composites: design, preparation, properties, and mechanisms. Mater. Sci. Eng. R.

[2] Song Y, Jiang F, Song N, Shi L, Ding P (2021). Multilayered structural design of flexible films for smart thermal management. Compos. Part A-Appl. Sci. Manufact..

[3] Zhu Z, Li C, Xie SEL, Geng R, Lin C (2019). Enhanced thermal conductivity of polyurethane composites via engineering small/large sizes interconnected boron nitride nanosheets. Compos. Sci. Technol..

[4] Huang C, Qian X, Yang R (2018). Thermal conductivity of polymers and polymer nanocomposites. Mater. Sci. Eng. R.

[5] Jiang F, Cui S, Rungnim C, Song N, Shi L (2019). Control of a dual-cross-linked boron nitride framework and the optimized design of the thermal conductive network for its thermoresponsive polymeric composites. Chem. Mater..

[6] Tong Z, Liu M, Bao H (2016). A numerical investigation on the heat conduction in high filler loading particulate composites. Int. J. Heat Mass Tran..

[7] Akatsuka M, Takezawa Y (2003). Study of high thermal conductive epoxy resins containing controlled high-order structures. J. Appl. Polym. Sci..

[8] Jeong I, Kim CB, Kang D-G, Jeong K-U, Jang SG (2019). Liquid crystalline epoxy resin with improved thermal conductivity by intermolecular dipole-dipole interactions. J. Polym. Sci. Pol. Chem..

[9] Yang X, Zhong X, Zhang J, Gu J (2021). Intrinsic high thermal conductive liquid crystal epoxy film simultaneously combining with excellent intrinsic self-healing performance. J. Mater. Sci. Technol..

[10] Yang X, Zhu J, Yang D, Zhang J, Guo Y (2020). High-efficiency improvement of thermal conductivities for epoxy composites from synthesized liquid crystal epoxy followed by doping BN fillers. Compos. Part B-Eng..

[11] Yu C, Zhang J, Li Z, Tian W, Wang L (2017). Enhanced through-plane thermal conductivity of boron nitride/epoxy composites. Compos. Part A-Appl. Sci. Manufact..

[12] Jiang F, Cui S, Song N, Shi L, Ding P (2018). Hydrogen bond-regulated boron nitride network structures for improved thermal conductive property of polyamide-imide composites. ACS Appl. Mater. Interfaces.

[13] Zha X, Yang J, Pu J, Feng C, Bai L (2019). Enhanced thermal conductivity and balanced mechanical performance of PP/BN composites with 1 vol% finely dispersed MWCNTs assisted by OBC. Adv. Mater. Interfaces.

[14] Zou D, Huang X, Zhu Y, Chen J, Jiang P (2019). Boron nitride nanosheets endow the traditional dielectric polymer composites with advanced thermal management capability. Compos. Sci. Technol..

[15] Han Y, Shi X, Yang X, Guo Y, Zhang J (2020). Enhanced thermal conductivities of epoxy nanocomposites *via* incorporating *in-situ* fabricated hetero-structured SiC-BNNS fillers. Compos. Sci. Technol..

[16] Guo H, Wang Q, Liu J, Du C, Li B (2019). Improved interfacial properties for largely enhanced thermal conductivity of poly(vinylidene fluoride)-based nanocomposites via functionalized multi-wall carbon nanotubes. Appl. Surf. Sci..

[17] Ma T, Zhao Y, Ruan K, Liu X, Zhang J (2020). Highly thermal conductivities, excellent mechanical robustness and flexibility, and outstanding thermal stabilities of aramid nanofiber composite papers with nacre-mimetic layered structures. ACS Appl. Mater. Interfaces.

[18] Ngo I-L, Vattikuti SVP, Byon C (2017). A modified Hashin-Shtrikman model for predicting the thermal conductivity of polymer composites reinforced with randomly distributed hybrid fillers. Int. J. Heat Mass Tran..

[19] Ruan K, Guo Y, Lu C, Shi X, Ma T (2021). Significant reduction of interfacial thermal resistance and phonon scattering in graphene/polyimide thermally conductive composite films for thermal management. Research.

[20] Agari Y, Uno T (1986). Estimation on thermal conductivities of filled polymers. J. Appl. Polym. Sci..

[21] Maxwell J (1873). Electricity and Magnetism.

[22] Nielsen LE (1973). Thermal conductivity of particulate-filled polymers. J. Appl. Polym. Sci..

[23] Li Y, Xu G, Guo Y, Ma T, Zhong X (2018). Fabrication, proposed model and simulation predictions on thermally conductive hybrid cyanate ester composites with boron nitride fillers. Compos. Part A-Appl. Sci. Manufact..

[24] Guo Y, Xu G, Yang X, Ruan K, Ma T (2018). Significantly enhanced and precisely modeled thermal conductivity in polyimide nanocomposites with chemically modified graphene via in situ polymerization and electrospinning-hot press technology. J. Mater. Chem. C.

[25] Guo Y, Ruan K, Yang X, Ma T, Kong J (2019). Constructing fully carbon-based fillers with a hierarchical structure to fabricate highly thermally conductive polyimide nanocomposites. J. Mater. Chem. C.

[26] Guo Y, Yang X, Ruan K, Kong J, Dong M (2019). Reduced graphene oxide heterostructured silver nanoparticles significantly enhanced thermal conductivities in hot-pressed electrospun polyimide nanocomposites. ACS Appl. Mater. Interfaces.

[27] Shi X, Zhang R, Ruan K, Ma T, Guo Y (2021). Improvement of thermal conductivities and simulation model for glass fabrics reinforced epoxy laminated composites *via* introducing hetero-structured BNN-30@BNNS fillers. J. Mater. Sci. Technol..

[28] Wang Z, Yang M, Cheng Y, Liu J, Xiao B (2019). Dielectric properties and thermal conductivity of epoxy composites using quantum-sized silver decorated core/shell structured alumina/polydopamine. Compos. Part A-Appl. Sci. Manufact..

[29] Gu J, Xie C, Li H, Dang J, Geng W (2014). Thermal percolation behavior of graphene nanoplatelets/polyphenylene sulfide thermal conductivity composites. Polym. Compos..

